# Titanium plate fixation versus conventional approach in the treatment of deep sternal wound infection

**DOI:** 10.1186/s13019-016-0458-3

**Published:** 2016-04-08

**Authors:** Wei Wang, Shaohua Wang

**Affiliations:** Division of Cardiac Surgery, Mazankowski Alberta Heart Institute, University of Alberta, Edmonton, AB Canada

**Keywords:** Sternum, Wound infection

## Abstract

**Background:**

Deep sternal wound infection (DSWI) is a serious complication post cardiac surgery and associated with increased mortality, morbidity and cost. Sternal titanium plate fixation could be an effective approach to treat DSWI. We sought to compare the effectiveness of titanium plate fixation with conventional approach in the treatment of DSWI.

**Methods:**

Retrospective data was analyzed from consecutive patients with DSWI post cardiac surgery who received either titanium plate fixation (sternal plate group) or conventional treatment with sternal debridement and rewiring (control group). Pre-operative risk factors and post-operative clinical outcome were compared between the 2 groups.

**Results:**

A total of 36 patients (mean age 65.0 ± 8.6, 63.9 % male) with DSWI were in the sternal plate group whereas 26 patients (mean age 64.0 ± 13.4, 65.4 % male) were in the control group. The mean follow-up period was 15.92 months. The major pre-operative comorbidities were comparable between the 2 groups. The rate of receiving multiple debridement procedures (≥3) was significantly lower in the sternal plate group (5.6 % vs. 26.9 %, *P* = 0.03). Patients in the sternal plate group had no treatment failure, whereas 42.3 % of patients in the control group had treatment failure requiring muscle flaps reconstruction by plastic surgery (0 % vs. 42.3 %, *P* < 0.001). There was a trend of lower in-hospital mortality (11.1 % vs. 19.2 %, *P* = 0.47) in the sternal plate group.

**Conclusion:**

Compared to conventional treatment, titanium plate fixation appears to have favorable clinical outcome.

## Background

Median sternotomy remains the most commonly used incision in patients undergoing cardiac surgery. Deep sternal wound infection (DSWI) is a serious complication post sternotomy. Although rates of DSWI are relatively low (range 0.4 to 5.1 %) [1–3], it is associated with higher mortality and morbidities, prolonged hospital stay, and increased patient suffering and cost [4, 5]. Conventional treatment of DSWI includes wound debridement, wound vacuum therapy (VAC) and sternal rewiring [6]. However, dehisced and infected sternums are sometimes very fragile that rewiring may not work, especially in patients with multiple co-morbidities. Plastic surgery is often consulted for chest wall reconstruction if rewiring fails to stabilize the sternum [7].

Titanium sternal plate fixation is a relatively new approach to treat DSWI. Early reports demonstrated good clinical outcomes [8–10]. However, most of previous studies were case series, and there was no comparison between sternal plate fixation and conventional treatment. In the current study, we sought to report our experience of sternal plate fixation in patients with DSWI post cardiac surgery, and to compare the clinical outcome between these two approaches.

## Methods

Retrospective data were analyzed from consecutive patients with DSWI post cardiac surgery who received titanium plate fixation (sternal plate group) from November 2008 to July 2013 at Mazankowski Alberta Heart Institute, University of Alberta. As comparison, consecutive patients with DSWI who received conventional treatment from January 2006 to October 2008 at the same heart institute before the titanium plate was introduced were identified as the control group. Pre-operative co-morbidities, post-operative mortality, length of hospital stay (LOS), and recurrence rates of infection were compared between the 2 groups. The study was approved by the Health Research Ethics Board, University of Alberta.

**Fig. 1:**
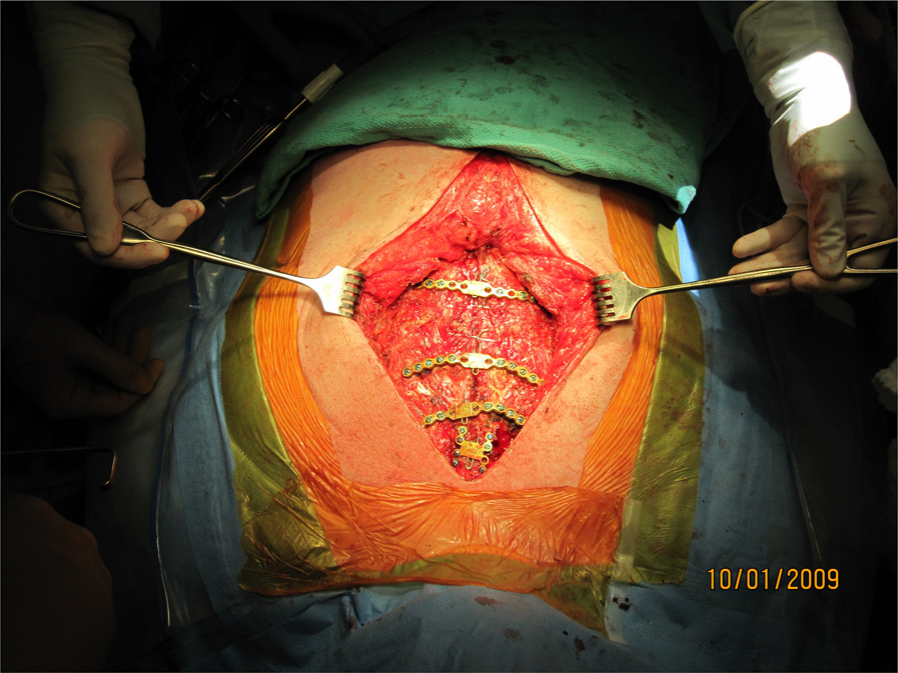
Illustration of titanium plate fixation. Detailed legend: Three transverse sternal plates and one “H” shape plate were used for sternal fixation

Preoperative blood cultures and wound swab were conducted routinely for all the patients. Antibiotics were given intravenously according to sensitivity results by infectious disease team. In both groups, surgical wound debridement followed by wound VAC therapy was routinely used. During debridement, all previous sternal wires were removed and wound edges including skin and subcutaneous tissue were excised until the healthy tissue was visible. A thin layer (1 mm) of bone was taken out along both sternal edges with a sternal saw. Wound VAC was set at negative pressure of 25–50 mmHg and changed 3 times a week. In the control group, sternal rewiring was performed if applicable. Plastic surgery was consulted when rewiring had failed. In the sternal plate group, patients were deemed ready for sternal plate fixation when good granulation was observed with no signs of local infection. All sternal plate fixation procedures were performed under general anesthesia. Bilateral composite muscular-cutaneous flaps were developed to expose the sternum and ribs to the midclavicular line. The Synthes Titanium Sternal Fixation System was used. We first reduced the sternum by using reduction forceps on both the superior and inferior aspects of the sternum. Next, we contoured the templates to the sternum and ribs, followed by contouring the titanium plates to match the templates. After anchoring the plates against the sternum and ribs and confirming they had the correct shape, 8 to 10 screws were screwed into the sternum and ribs. Three to four transverse titanium plates were placed to achieve complete sternal integrity. One “H” shape short plate was often used at the top aspect of the sternum as very limited muscular-cutaneous flaps could develop in this area (Fig. 1). Bilateral small Hemovac drains were placed between the sternal plates and the composite flaps. The composite flaps were brought together with interrupted No. 1 Vicryl (Ethicon Inc.) sutures. Skin was closed with interrupted 3-0 prolene sutures. Patients in both groups received intravenous antibiotics for a course of 6 weeks after the last procedure.

Continuous data were reported as means ± standard deviations (SD) and were compared by Student’s t-test. Categorical data were reported as proportions and compared using χ2 test. *P* value of <0.05 was adopted as statistical significant.

## Results

There were 36 patients (mean age 65.0 ± 8.6, 63.9 % male) in the titanium plate fixation group and 26 patients (mean age 64.0 ± 13.4, 65.4 % male) in the control group. Demographic and pre-operative data were shown in Table 1. The pre-operative comorbidities including hypertension, hyperlipidemia, diabetes, body mass index, smoking, chronic obstructive pulmonary disease (COPD), chronic renal dysfunction and left ventricular ejection fraction were all comparable between the 2 groups.

**Table 1:** Pre-operative comobidities

	Sternal plate group (*n* = 36)	Control group (*n* = 26)	*P* value
Male	63.9 %	65.4 %	0.90
Mean age (year)	65.0 ± 8.6	64.0 ± 13.4	0.71
HTN	83.3 %	92.3 %	0.30
Hyperlipidemia	66.7 %	73.1 %	0.59
DM	55.6 %	53.8 %	0.89
Smoking	50.0 %	38.5 %	0.37
COPD	30.6 %	26.9 %	0.76
Obesity BMI	52.8 %	46.2 %	0.61
Renal failure	30.6 %	11.5 %	0.08
LVEF	46.2 % ± 13.0 %	45.0 % ± 11.8 %	0.75

*Abbreviations*: *HTN* hypertension, *DM* diabetes mellitus, *COPD* chronic obstructive pulmonary disease, *BMI* body mass index, *LVEF* left ventricular ejection fraction

Post-operative clinical outcome was shown in Table 2. Patients in the sternal plate group had a significantly lower rate of having multiple (3 times or greater) debridement procedures than patients in the control group (5.6 % vs. 26.9 %, *P* = 0.03). All patients in the sternal plate group had stable sternum, whereas 42.3 % of patients in the control group had treatment failure with unstable sternum and required muscle flaps reconstruction by plastic surgery (0 % vs. 42.3 %, *P* < 0.001). Two patients in the sternal plate group had failed rewiring previously. Follow-up time was 6 to 47 months (mean 15.92 months) for all patients. Although the mortality was much less in the sternal plate group (11.1 %) than in the control group (19.2 %), there was no statistical significant difference between the 2 groups (*P* = 0.47). One patient in the sternal plate group and one patient in the control group died from sepsis and other deaths were not related to DSWI. The hospital length of stay (LOS) was comparable between the 2 groups (48.0 ± 46.0 vs. 57.4 ± 53.2 days, respectively, *P* = 0.498). Five patients (13.9 %) in sternal plate group and 5 patients (19.2 %) in the control group had recurrent superficial sternal infection. Among those patients, two (5.6 %) in the sternal plate group had plate removal and one (3.8 %) in the control group had wires removal due to chronic draining sinus.

**Table 2:** Post-operative outcome

	Sternal plate group (*n* = 36)	Control group (*n* = 26)	*P* value
In hospital death	11.1 %	19.2 %	0.47
Treatment failure	0 %	42.3 %	<0.001
Multiple debridements (≥3)	5.6 %	26.9 %	0.02
Recurrent DSWI	0	0	N/A
Recurrent superficial infection	13.9 %	19.2 %	0.57
Plate/wire removal	5.6 %	3.8 %	0.76
Hospital length of stay (d)	48.0 ± 46.0	57.4 ± 53.2	0.50

Treatment failure: Unstable sternum post either sternal rewiring or chest wall fixation

*Abbreviations*: *DSWI* deep sternal wound infection

## Discussion

Studies have shown that risk factors of DSWI post cardiac surgery include prolonged intubation, bilateral internal mammary arteries use, diabetes, post-op bleeding, high body mass index (BMI) and combined surgery [4]. Wound debridement, infection control and achieving integrity of sternum by rewiring are the mainstays in the management of DSWI. However, sternal rewiring often fails to achieve sternum healing and stability due to poor sternum quality and multiple fractures, especially in patients who have advanced age, obesity, diabetes and COPD. Failed rewiring can lead to partial or complete sternum removal and consequent sternal defects [2]. Although not reported very often, the rate of primary rewiring failure can be as high as 45 % [11]. Our study showed similar results that even after multiple debridement procedures, 42.3 % of patients in the conventional treatment group had failure of rewiring and required plastic surgery interventions. Titanium sternal plate fixation is an effective and novel approach to stabilize the sternum in patients with severe DSWI. By pulling both the sternum and the ribs together, the titanium plates have much stronger force to stabilize the whole chest wall. It is important to note that rib-to-rib plating does not provide consistent results. It is better to salvage sternal fragments, if they are viable and use them as anchoring points. In our study, none of the patients who had received sternal plate fixation had sternum instability. Indeed, 2 of the patients in the sternal plate group were initially treated with rewiring but failed. Sternal plates can also be used in patients who had nonunion sternum post cardiac surgery without sternal infection, especially when patients are in immunosuppressive status.

Wound VAC system is an important supplementary approach for DSWI management. The negative pressure provided by wound VAC can reduce wound edema and improve wound granulation and healing. However, wound VAC alone did not decrease total hospital stay [12], possibly because the sternum was still unstable. Studies have shown that wound VAC application followed by titanium sternal plating was a good strategy to treat DSWI post cardiac surgery [13, 14]. To date, the ideal duration of wound VAC therapy prior to sternal plate application is not well defined. The reported mean wound VAC therapy time varied from 2 to 5 weeks. Despite prolonged wound VAC time, the recurrence rates of sternal infection were still high (9.8 to 18.2 %). Gaudreau *et al* suggested that the criteria for switching wound VAC to sternal plate fixation included: granulation tissue appeared in the wound; repeated negative sternal wound cultures and low C-reactive protein levels [14]. In our series, the decision making to switch wound VAC to sternal plate fixation was based on clinical assessment. Once the wound looked healthy with good granulation and there were no signs of systemic or local infection, sternal plate fixation was carried out. Although the mean duration of wound VAC before sternal plate fixation in our study was relatively short (9.35 ± 9.99 days), there was no recurrence of DSWI and the rate of recurrent superficial sternal infection (13.9 %) was not higher than others. Shortened wound VAC duration might translate into shorter LOS and therefore reduces cost.

One concern of the titanium plate is recurrence of infection after implanting large pieces of foreign body into an already infected chest wall. In our series, 2 patients (5.6 %) in the sternal plate group had plate removal due to recurrence of superficial infection. However, at the time of plate removal both patients had very stable and fused sternum and no evidence of recurrence of deep infection. Even though both patients required another surgery to take out the plates, the quality of life was much improved with lack of pain and breathing distress due to moving sternum.

Our study has several limitations. Firstly, this is a single center, retrospective cohort study. Although most surgeons may agree that sternal plate fixation is an effective approach for the treatment of DSWI post cardiac surgery, a randomized control trial is warranted to better compare this new approach with conventional treatment. Secondly, our current study did not perform cost efficient analysis. The titanium plate system is expensive. However, it may have reduced the cost since patients required less aggressive procedures.

## Conclusion

In conclusion, titanium plate fixation appeared to be an effective method in the treatment of DSWI post cardiac surgery. Compared to conventional treatment, sternal plate fixation is associated with less debridement procedures and treatment failure.
